# Impact of Printing Orientation and Printer Type on the Accuracy of 3D-Printed Dental Splints

**DOI:** 10.3390/ma19112266

**Published:** 2026-05-27

**Authors:** Mohammed Hammamy, Neeraj Surathu, Nitish Surathu, Nathaniel C. Lawson, Andrea C. Dardashtian

**Affiliations:** 1 Division of Biomaterials, School of Dentistry, University of Alabama at Birmingham, Birmingham, AL 35209, USA; nlawson@uab.edu; 2 Georgia School of Orthodontics, Atlanta, GA 30350, USA; adardashtian@gaorthodontics.org; 3 The ACE Institute, Hamilton 3210, New Zealand; neeraj.surathu24@gmail.com (N.S.);

**Keywords:** occlusal splints, 3D-printing, DLP printing, LCD printing, trueness, build orientation

## Abstract

Additive manufacturing has expanded the fabrication of occlusal splints; however, the influence of printing technology and build orientation on accuracy remains unclear. This study evaluated the effect of printing technology (Liquid Crystal Display [LCD] and Digital Light Processing [DLP]) and build orientation (0°, 45°, and 90°) on the trueness of additively manufactured occlusal splints under standardized conditions. A maxillary occlusal splint was digitally designed from a scanned typodont and used as the reference model. Specimens (n = 10/group) were fabricated using two LCD printers (Ackuretta SOL, Phrozen Mini 8K S) and one DLP printer (SprintRay Pro 95S) with a single photopolymer resin (KeySplint Soft). All samples were printed at 100 µm layer thickness and subjected to standardized post-processing. Trueness was assessed by comparing scanned splints to the reference STL using Geomagic Control X and expressed as root mean square (RMS) values. Data were analyzed using two-way ANOVA and Tukey post hoc tests (α = 0.05). Printing technology and build orientation significantly affected trueness (*p* < 0.001), with RMS values increasing as build orientation increased, and no significant interaction between factors (*p* > 0.05). LCD systems demonstrated lower RMS values than the DLP system across all orientations. Within the limitations of this study, both factors influenced trueness; however, all systems produced clinically comparable results, supporting the use of LCD technology as a cost-effective option for occlusal splint fabrication.

## 1. Introduction

Occlusal trauma is defined as the application of excessive occlusal forces that surpass the adaptive and reparative capacity of the periodontium, resulting in injury, and is commonly referred to as traumatic occlusion [1]. It may be broadly classified into acute and chronic forms. Acute occlusal trauma typically arises from abrupt excessive forces, such as those generated by biting on hard objects or from improperly adjusted restorations and prosthetic appliances [1]. In contrast, chronic occlusal trauma is more prevalent and develops progressively over time, often in association with occlusal wear, tooth migration, and parafunctional habits such as clenching and bruxism. Occlusal splints, also referred to as bite guards, are widely utilized in the management of occlusal trauma and temporomandibular disorders. These appliances serve multiple therapeutic functions, including reducing loading on the temporomandibular joint, protecting the dentition from excessive occlusal forces, and facilitating muscle relaxation [2].

Traditionally, occlusal splints have been fabricated using vacuum-forming or injection molding techniques with acrylic resins, relying on conventional physical impressions and stone casts [2]. Although these methods have demonstrated clinical effectiveness, they are inherently labor-intensive and involve multiple steps, including impression taking, cast fabrication, wax-ups, and processing of polymethyl methacrylate, each of which may introduce potential sources of error and variability [3,4,5]. Moreover, conventionally fabricated splints have been associated with disadvantages such as thermal discomfort, unpleasant taste, residual monomer content, and dimensional instability [4,5].

Advancements in digital dentistry have substantially expanded splint fabrication through computer-aided design and manufacturing technologies, including both subtractive (milling) and additive (three-dimensional printing) approaches [2,3]. While subtractive manufacturing removes material from prefabricated solid blocks and can result in considerable material waste [6], additive manufacturing fabricates objects layer by layer through photopolymerization of liquid resins, allowing more efficient and economical appliance production with reduced waste [3,6]. Initially introduced for dental cast fabrication, three-dimensional printing has since expanded to occlusal splints [7], offering advantages such as rapid production, enhanced customization, improved patient comfort, cost-effectiveness, and greater production efficiency compared to conventional and milled techniques [2,4,8,9], although printed splints have been reported to exhibit lower accuracy than milled splints [2].

Accuracy is a critical consideration in occlusal splint fabrication, as dimensional discrepancies at the intaglio surface may compromise retention, alter occlusal contacts, and increase the need for chairside adjustments [10,11,12]. Deviations beyond clinically acceptable thresholds may negatively affect fit, occlusion, and treatment outcomes. The accuracy of additively manufactured splints is influenced by multiple factors, including printing technology, material properties, layer thickness, post-processing protocols, and build orientation [6,13]. Among these, printing technology and build orientation are key determinants of dimensional accuracy and mechanical performance.

Among additive manufacturing technologies, vat photopolymerization technologies, including stereolithography (SLA), digital light processing (DLP), and liquid crystal display (LCD), are the most widely utilized in dentistry [3]. SLA employs a focused ultraviolet laser, DLP polymerizes entire layers simultaneously using a projector, LCD uses a masked light source to cure complete layers in a single exposure [6,14]. These differences in light source and image projection mechanisms influence the polymerization process and dimensional accuracy of printed objects [6,14].

Comparative investigations have reported technology-dependent differences in trueness and precision, with some studies favoring DLP systems, while others demonstrate minimal or clinically acceptable differences among technologies under standardized conditions [7,15,16]. Specifically for printed splints, a previous study reported greater accuracy for a DLP printer than an SLA printer [15]. Thus, controversy remains, as some authors report negligible clinical impact, whereas others identify measurable discrepancies that may influence clinical performance [6,17].

Additively manufactured polymers exhibit anisotropic behavior as a result of their layer-by-layer fabrication process, leading to direction-dependent material properties [13]. Printing splints in a horizontal orientation (0°) minimizes the number of printed layers and interlayer interfaces, which may reduce dimensional discrepancies and improve overall accuracy [18]. Printing in a horizontal orientation also provides the smoothest surface which could also affect fit [13]. Most previous studies have reported improved accuracy of printed splints when printed at lower build angles [6,10,15,17,18]. Higher build angles, however, have been shown to improve mechanical properties [18,19,20,21].

Although LCD technology has gained popularity due to its affordability and accessibility, limited evidence exists evaluating its accuracy compared to established systems such as DLP, particularly in occlusal splint fabrication [7,13]. Therefore, the aim of this study was to evaluate the effect of printing technology (LCD versus DLP) and build orientation (0°, 45°, and 90°) on the trueness of additively manufactured occlusal splints under standardized conditions. The null hypotheses were that neither printing technology nor build orientation would significantly affect the trueness of additively manufactured occlusal splints as measured by root mean square (RMS) deviation.

## 2. Materials and Methods

An upper arch occlusal splint was designed from a digitally scanned dental typodont using splint design software (Medit Splints (v1.1.0); Medit Corp., Seoul, Republic of Korea). The final design was exported as a standard tessellation language (STL) file and used as the reference model for all comparisons.

Splints were fabricated using three vat photopolymerization three-dimensional (3D) printers, including two liquid crystal display (LCD) systems (Ackuretta SOL, Ackuretta, Taipei City, Taiwan; (Phrozen Mini 8K S, Phrozen Technology, Hsinchu City, Taiwan) with a Ferguson-Desai Double Arch Stealth Plate (build platform) and one digital light processing (DLP) system (SprintRay Pro 95S, SprintRay Inc., Los Angeles, CA, USA). The STL file was imported into the respective printer software for processing (ALPHA AI (v5.1.3) for Ackuretta, Chitubox (v2.3.1) for Phrozen, and RayWare (v2.9) for SprintRay). All specimens were printed using the same photopolymer resin (KeySplint Soft; Keystone Industries, Gibbstown, NJ, USA) to minimize material-related variability.

**Figure 1:**
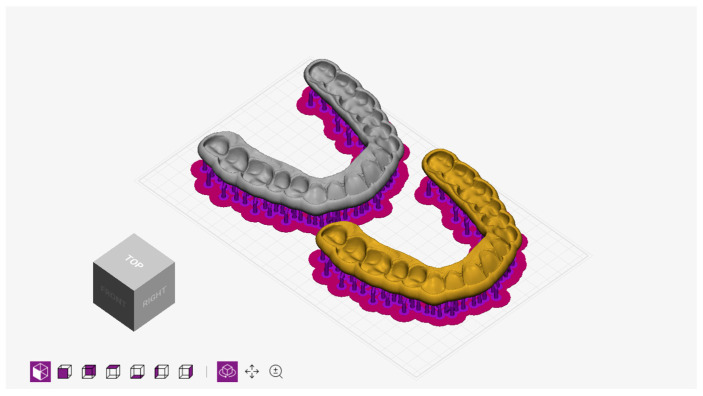
Representative occlusal splints positioned in a 0° (horizontal) orientation relative to the build platform with support structures attached to the cameo surface.

**Figure 2:**
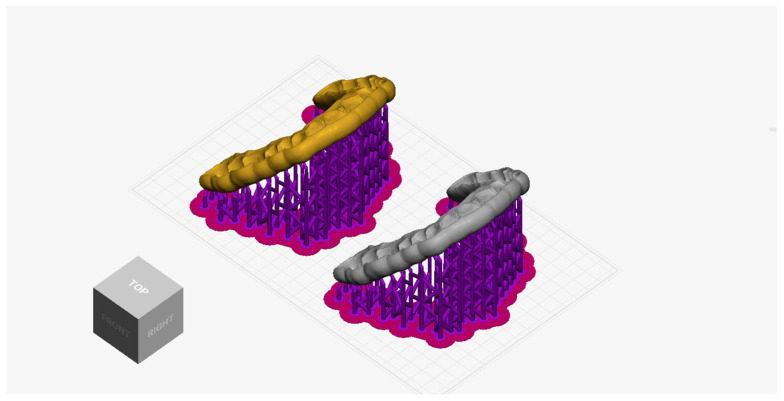
Representative occlusal splints positioned in a 45° orientation relative to the build platform with support structures attached to the cameo surface.

**Figure 3:**
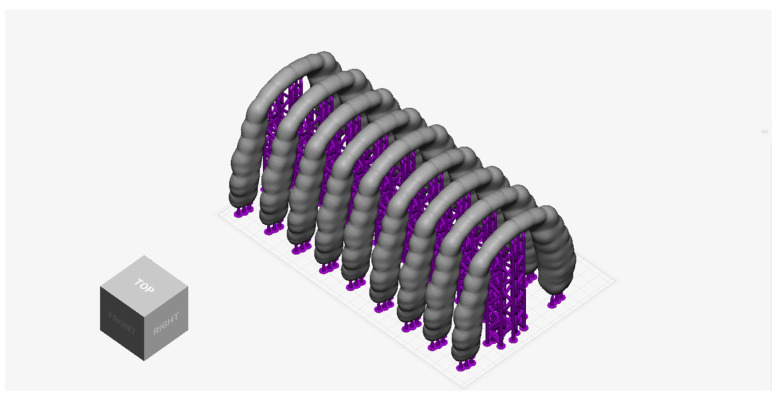
Representative occlusal splints positioned in a 90° (vertical) orientation relative to the build platform with support structures attached to the cameo surface.

Each splint was fabricated at three build orientations relative to the build platform: (1) 0°, with the occlusal surface facing the build plate; (2) 45° inclination; and (3) 90° vertical orientation. All specimens were printed with support structures and a uniform layer thickness of 100 µm. A total of 10 specimens were fabricated per group (n = 10), resulting in nine experimental groups based on the combination of printing technology and build orientation. Representative screenshots are presented in Figure 1, Figure 2 and Figure 3.

Following fabrication, all specimens underwent standardized post-processing. Printed splints were washed in 91% isopropyl alcohol using a two-stage cleaning cycle in a Pro Wash/Dry unit (SprintRay Inc., Los Angeles, CA, USA) to remove uncured resin, followed by air drying. For all groups, post-curing was performed using a Curie Plus curing unit (Ackuretta, Taiwan) according to the manufacturer’s recommended parameters for the resin material. The same curing unit was used for all groups to eliminate this sources of variability and isolate the effects of the printing technology.

Trueness was evaluated by comparing each printed splint to the original reference STL file. All specimens were digitized using a laboratory scanner (3Shape E4; 3Shape, Copenhagen, Denmark). The scanned files were imported into three-dimensional inspection software (Geomagic Control X (v2024.2.0); 3D Systems, Rock Hill, SC, USA). Each test model was aligned to the reference STL using a best-fit alignment algorithm. A three-dimensional deviation analysis was performed, and root mean square (RMS) values were calculated to quantify discrepancies between the printed and reference models, with lower RMS values indicating higher trueness.

Statistical analysis was performed using statistical software (SPSS (v29.0); IBM Corp., Armonk, NY, USA). A two-way analysis of variance (ANOVA) was conducted to evaluate the effects of printing technology and build orientation on RMS values, as well as their interaction. Post hoc comparisons were performed using Tukey’s honestly significant difference (HSD) test. Statistical significance was set at α = 0.05.

## 3. Results

Root mean square (RMS) values were used to evaluate the trueness of occlusal splints fabricated using three printing systems at build orientations of 0°, 45°, and 90°. Descriptive statistics are presented in Table 1.

**Figure 4:**
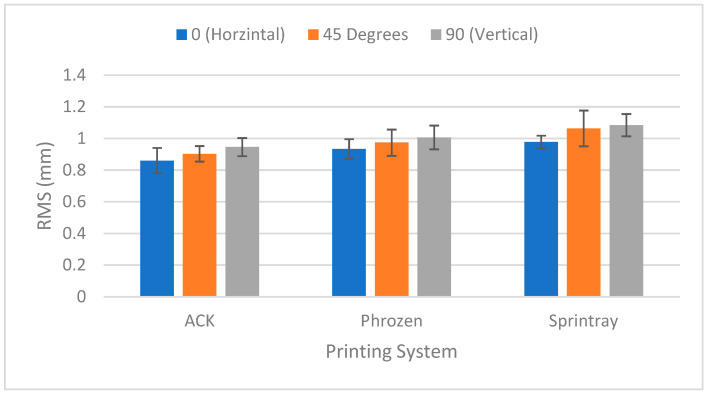
Mean RMS values (mm) of occlusal splints across printing technologies and build orientations.

**Figure 5:**
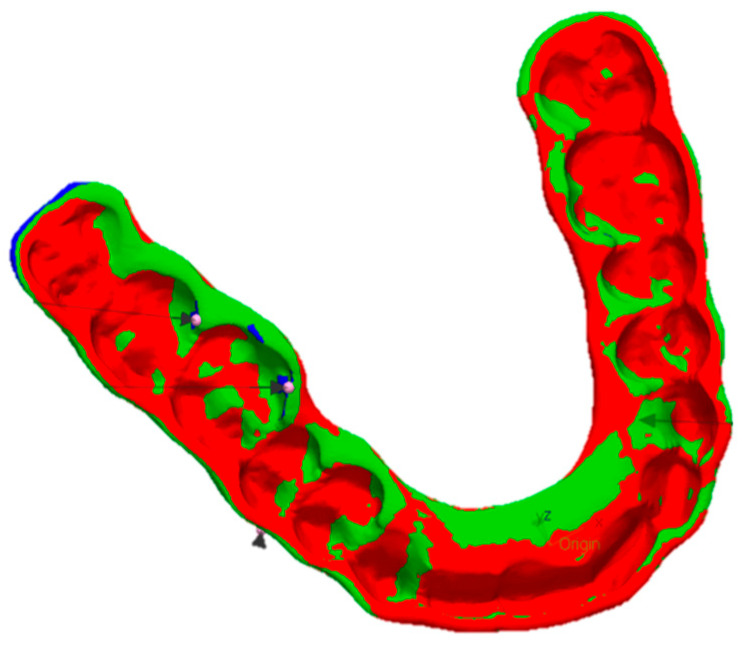
Representative color deviation map of a 3D-printed occlusal splint compared to the reference STL model, analyzed using Geomagic Control X. Positive deviations are shown in red, negative deviations in blue, and minimal deviations in green.

Across all printing systems, RMS values increased with increasing build orientation (Figure 4). The ACK system demonstrated mean RMS values of 0.8599 at 0°, 0.9029 at 45°, and 0.9452 at 90°. The Phrozen system showed mean values of 0.9334, 0.9735, and 1.0066, while the SprintRay system exhibited the highest RMS values across all orientations, with means of 0.9776, 1.0634, and 1.0839 at 0°, 45°, and 90°, respectively. Representative color deviation maps illustrating surface discrepancies between printed splints and the reference model are shown in Figure 5.

A two-way analysis of variance (ANOVA) demonstrated that both printing technology and build orientation had a statistically significant effect on RMS values. Printing technology showed a significant main effect (F = 27.320, *p* < 0.001), as did build orientation (F = 11.308, *p* < 0.001). No significant interaction was observed between printing technology and build orientation (F = 0.331, *p* = 0.857). The overall model was statistically significant (F = 9.822, *p* < 0.001) and explained approximately 49.2% of the variance in RMS values (R^2^ = 0.492).

Post hoc analysis using Tukey’s honestly significant difference (HSD) test revealed no statistically significant pairwise differences between individual build orientations (*p* > 0.05), despite a consistent increasing trend from 0° to 90°.

**Figure 6:**
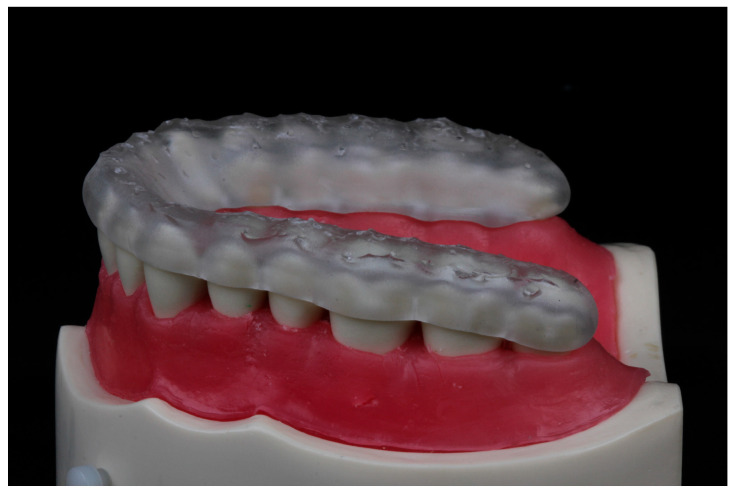
Representative 0-printed splint on typodont model. Remnants of supports on cameo were not removed as the cameo surfaces was not included in the analysis.

**Figure 7:**
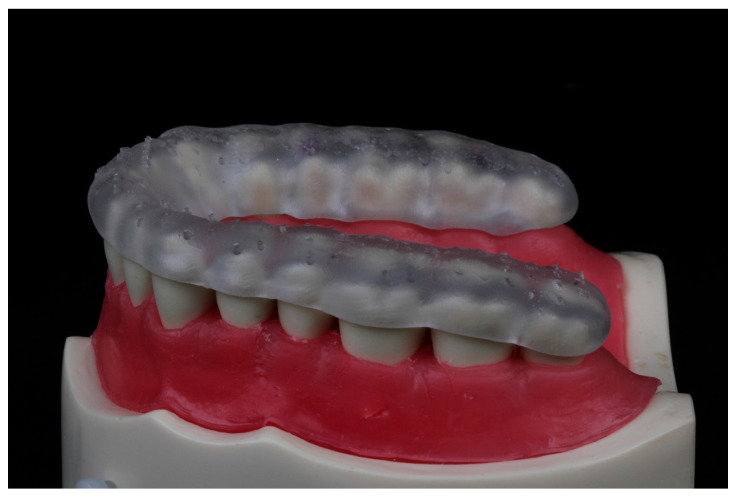
Representative 45-printed splint on typodont model. Remnants of supports on cameo were not removed as the cameo surfaces was not included in the analysis.

**Figure 8:**
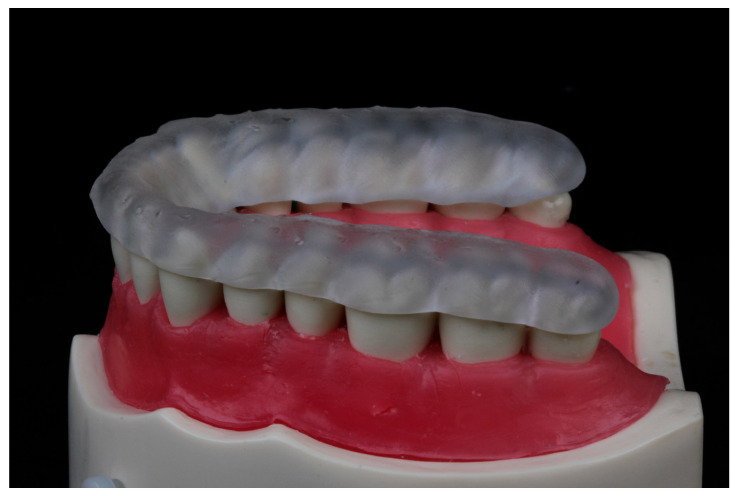
Representative 90-printed splint on typodont model. Remnants of supports on cameo were not removed as the cameo surfaces was not included in the analysis.

Photographs of representative splints at 0°, 45° and 90° are presented in Figure 6, Figure 7 and Figure 8.

## 4. Discussion

The present study evaluated the influence of printing technology and build orientation on the trueness of additively manufactured occlusal splints under standardized conditions. The findings demonstrated that both printing technology and build orientation significantly affected root mean square (RMS) values, while no significant interaction effect was observed between these variables.

Build orientation exhibited a consistent influence on trueness across all printing systems, with RMS values increasing as the orientation angle increased from 0° to 90°. Although post hoc analysis did not reveal statistically significant pairwise differences between individual orientations, the observed distribution of means, as reflected in the Tukey homogeneous subsets, indicated a tendency toward lower RMS values at 0° orientation compared to higher build angles. These findings are consistent with previous studies reporting that reduced build angles may minimize layer-induced distortion and improve surface fidelity [10,17]. This phenomenon may be explained by the layer-by-layer fabrication process inherent to additive manufacturing, in which incremental deviations occur at the interface of each deposited layer and may accumulate, contributing to overall dimensional inaccuracy. At lower build orientations, the printed layers are more closely aligned with the geometry of the object, thereby reducing the “staircase effect” and limiting cumulative distortion. In contrast, higher build angles increase the number of layer interfaces and frequently require additional support structures, both of which can contribute to greater surface irregularities and dimensional deviations [5,20,21].

The absence of a significant interaction between printing technology and build orientation suggests that the effect of orientation on trueness is independent of the printing system used. This suggests that the influence of build orientation is governed primarily by geometric and material-related factors inherent to the additive manufacturing process, rather than by the specific light-curing mechanism of the printer. Consequently, optimization of build orientation may be broadly applicable across different vat photopolymerization technologies. From a clinical perspective, this finding supports the selection of lower build orientations to enhance dimensional accuracy regardless of the printing technology used.

Printing technology was also found to have a statistically significant effect on trueness. In the present study, the LCD-based systems demonstrated lower RMS values compared to the DLP system evaluated. Specifically, both LCD-based printers (ACK and Phrozen) consistently exhibited lower mean RMS values across all build orientations compared to the DLP (SprintRay), indicating improved overall trueness under the conditions investigated. This finding contrasts with a previous investigation that reported superior trueness for DLP systems [7]. The findings of this study therefore suggest that contemporary LCD-based technologies may achieve comparable, or in some cases superior, dimensional accuracy relative to DLP systems, particularly when optimized printing parameters and standardized workflows are employed.

Several factors may account for this observation. LCD-based systems utilize a masked light source that enables uniform layer-wise polymerization, thereby potentially enhancing consistency across the build platform. In contrast, DLP systems rely on projected light patterns, which may introduce variations in light intensity distribution across the projection field and subsequently affect polymerization uniformity and dimensional accuracy. Additionally, discrepancies between studies may be attributed to variations in printer calibration, exposure settings, and build platform configurations, as well as differences in post-processing protocols such as washing and post-curing conditions [6,13].

It is important to note, however, that while statistically significant differences were seen between printing technologies, the magnitude of these differences should be interpreted within a clinical context. Previous literature has suggested that variations in trueness among additive manufacturing systems may fall within clinically acceptable thresholds, particularly when standardized protocols are followed [15,16]. Therefore, although the present findings of this study highlight a measurable advantage of LCD-based systems in terms of RMS values, both LCD and DLP technologies may be capable of producing clinically acceptable occlusal splints.

Collectively, these findings support the growing body of evidence indicating that LCD-based 3D printing represents a viable and cost-effective alternative to DLP systems for the fabrication of occlusal splints, with the potential to expand access to digital workflows without compromising accuracy.

The influence of build orientation on trueness may also be explained by the anisotropic behavior of additively manufactured polymers. Due to the layer-by-layer fabrication process, mechanical and dimensional properties vary depending on direction, resulting in anisotropic material behavior [13]. At higher build orientations, increased layer stacking along the vertical axis may introduce weak interlayer bonding and increased susceptibility to distortion, particularly in geometrically complex structures such as occlusal splints. This directional dependence of material properties may contribute to variations in flexural strength, wear resistance, and dimensional stability, thereby influencing overall trueness [5,20].

Although statistically significant main effects were identified for build orientation, post hoc analysis did not reveal significant pairwise differences between individual orientations. The findings of this study therefore suggest that while build orientation contributes to overall variability in trueness, the magnitude of these differences between specific orientations may be limited. These findings are consistent with previous studies indicating that variations in printing parameters may not always result in clinically significant differences when standardized fabrication protocols are employed [15,16].

The findings of this study have important clinical implications. The comparable or improved performance of LCD-based systems suggests that cost-effective and accessible printing technologies may provide clinically acceptable accuracy for the fabrication of occlusal splints. This has the potential to expand the inclusion of in-office additive manufacturing workflows, facilitating reduced production times, improved efficiency, and increased accessibility to digital treatment modalities [8,9].

Several limitations of this study should be acknowledged. First, the investigation was conducted under in vitro conditions, which may not fully replicate intraoral clinical environments. Second, only a single resin type and standardized printing parameters were evaluated, which may limit the generalizability of the findings [6]. Lastly, only trueness was assessed, and other clinically relevant properties such as wear resistance, mechanical strength, and long-term dimensional stability were not evaluated [13]. Future research should focus on evaluating the clinical performance of 3D-printed occlusal splints fabricated using different printing technologies and build orientations, as well as investigating the influence of material composition, post-processing protocols, and long-term intraoral use on splint accuracy and durability.

## 5. Conclusions

This study demonstrated that both printing technology and build orientation significantly influence the trueness of additively manufactured occlusal splints based on RMS values. While lower build orientations showed a consistent trend toward improved trueness, differences between individual orientations were not statistically significant. LCD-based systems exhibited comparable or superior trueness relative to the DLP system evaluated, suggesting that affordable LCD technology may represent a permissible alternative for splint fabrication. Within a clinical context, both LCD and DLP systems produced trueness values within a comparable range, indicating their suitability for clinical application. These findings support the integration of cost-effective additive manufacturing technologies into digital workflows without compromising accuracy.

## Figures and Tables

**Table 1:** Mean RMS values (mm) of occlusal splints by printing technology and build orientation.

Orientation	ACK (LCD)	Phrozen (LCD)	SprintRay (DLP)
0°	0.86 ± 0.08	0.933 ± 0.062	0.978 ± 0.04
45°	0.903 ± 0.049	0.974 ± 0.083	1.063 ± 0.113
90°	0.945 ± 0.057	1.007 ± 0.075	1.084 ± 0.07

## Data Availability

The original contributions presented in this study are included in the article. Further inquiries can be directed to the corresponding author.

## Abbreviations

The following abbreviations are used in this manuscript:

RMS	Root mean square
DLP	Digital light processing
LCD	Liquid crystal display
TMJ	Temporomandibular joint
CAD/CAM	Computer-aided design and computer-aided manufacturing
3D	Three-dimensional
STL	Standard tessellation language
PMMA	Polymethyl methacrylate
SLA	Stereolithography
ANOVA	Analysis of variance
HSD	Honestly significant difference
